# The social responsiveness scale is an efficient screening tool for autism spectrum disorder traits in adults with anorexia nervosa

**DOI:** 10.1002/erv.2736

**Published:** 2020-04-03

**Authors:** Jess Kerr‐Gaffney, Amy Harrison, Kate Tchanturia

**Affiliations:** ^1^ Department of Psychological Medicine Institute of Psychiatry, Psychology, and Neuroscience, King's College London London UK; ^2^ Department of Psychology and Human Development University College London London UK; ^3^ South London and Maudsley NHS Trust, National Eating Disorders Service Psychological Medicine Clinical Academic Group London UK; ^4^ Department of Psychology Ilia State University Tbilisi Georgia

**Keywords:** anorexia nervosa, autism spectrum disorder, clinical interview, comorbidity, self‐report

## Abstract

**Objective:**

A significant proportion of individuals with anorexia nervosa (AN) show high levels of autism spectrum disorder (ASD) traits, a factor associated with poorer treatment outcomes. An important question for both researchers and clinicians relates to how ASD traits should be assessed in individuals with AN. This study aimed to examine scores on the Social Responsiveness Scale adult self‐report version (SRS‐2) in individuals in the acute (AN) and recovered stages (REC) of illness compared to healthy controls (HCs). We also aimed to examine associations between the SRS‐2 and an observational diagnostic measure, the Autism Diagnostic Observation Schedule ‐ second edition (ADOS‐2).

**Method:**

The SRS‐2 and ADOS‐2 were administered to 142 adults with AN, REC, and HCs. Eating disorder (ED) psychopathology and functional impairment were also assessed.

**Results:**

AN and REC scored significantly higher than HCs on the SRS‐2. SRS‐2 scores significantly predicted ADOS‐2 classification and were positively associated with ED psychopathology and functional impairment. SRS‐2 scores were not associated with BMI or illness duration.

**Conclusions:**

The SRS‐2 may be a useful tool in screening for ASD traits in individuals with AN. Although cross‐sectional, the results also suggest ASD symptoms are independent of BMI and persist in individuals recovered from AN.

**Highlights**

- Individuals in the acute and recovered stage of illness show higher levels of ASD traits than healthy controls on both self‐report and clinical interview measures
- Score on the social responsiveness scale, adult self‐report version (SRS‐2) are associated with scores on the Autism Diagnostic Observation Schedule, second edition (ADOS‐2), suggesting the SRS‐2 may be useful screening tool for ASD in individuals with AN
- ASD traits were positively associated with severity of eating disorder psychopathology and functional impairment, but not BMI or illness duration

## 1 INTRODUCTION

Over the past few decades, research has accumulated suggesting a relationship between anorexia nervosa (AN) and autism spectrum disorder (ASD). AN is a severe and potentially life‐threatening eating disorder (ED) characterised by persistent restriction of energy intake and a disturbance in the way in which one's body weight or shape is experienced. In contrast, ASD is a neurodevelopmental disorder characterised by deficits in social communication and interaction and restrictive, repetitive patterns of behaviour or interests (American Psychiatric Association, 2013). While AN affects primarily females and typically develops in adolescence (Herpertz‐Dahlmann, van Elburg, Castro‐Fornieles, & Schmidt, 2015), ASD is more commonly diagnosed in males and symptoms are present from early childhood (Fombonne, 2009). Nonetheless, empirical research has documented a number of similarities in the phenotypic expressions of AN and ASD. In the neurocognitive domain, difficulties in set‐shifting, weak central coherence, and superior attention to detail are seen in both individuals with AN and ASD (Happé & Booth, 2008; Jolliffe & Baron‐Cohen, 1997; Lang, Lopez, Stahl, Tchanturia, & Treasure, 2014; Westwood, Stahl, Mandy, & Tchanturia, 2016), as well as their first‐degree relatives (Bölte & Poustka, 2006; Holliday, Tchanturia, Landau, Collier, & Treasure, 2005; Tenconi et al., 2010; Wong, Maybery, Bishop, Maley, & Hallmayer, 2006). Regarding social‐cognitive functioning, difficulties in theory of mind (ToM) and emotion recognition have been replicated extensively in individuals with ASD (Bal et al., 2010; Baron‐Cohen, Wheelwright, Hill, Raste, & Plumb, 2001; Happé, 1994; Kleinman, Marciano, & Ault, 2001; Kuusikko et al., 2009). Similarly, those with AN also show difficulties on ToM and facial emotion recognition tasks compared to healthy controls (HCs), although generally differences are of a smaller magnitude (Bora & Kose, 2016; Leppanen, Sedgewick, Treasure, & Tchanturia, 2018). Further, high levels of alexithymia and social anxiety are apparent in both disorders (Kerr‐Gaffney, Harrison, & Tchanturia, 2018; Kinnaird, Stewart, & Tchanturia, 2019; Spain, Sin, Linder, McMahon, & Happé, 2018; Westwood, Kerr‐Gaffney, Stahl, & Tchanturia, 2017).

Given these similarities, it is perhaps not surprising that between 4 and 52.5% of individuals with AN show clinically significant levels of ASD traits (Westwood & Tchanturia, 2017). This variation in part likely reflects differences in the tools used to assess ASD in those with AN. In order to provide a full diagnostic assessment of ASD, clinical guidelines recommend a formal assessment of current symptoms, using tools such as the Autism Diagnostic Observation Schedule (ADOS; Lord et al., 2000) as well as an assessment of early developmental history where possible (National Institute for Health and Clinical Excellence [NICE], 2012). However, this poses a problem for both researchers and clinicians; “gold standard” assessment tools such as the ADOS are lengthy, costly, and require extensive, ongoing training to administer. In addition, developmental history assessments such as the Autism Diagnostic Interview – Revised (ADI‐R; Lord, Rutter, & Le Couteur, 1994) require an interview with an informant (e.g., a parent or guardian), which may be difficult to obtain from the families of adults with AN. Therefore, brief screening methods that aid identification of possible cases are required. Such measures should correlate with more comprehensive assessment tools in order to be useful. Further, measures that show agreement with measures of adaptive behaviour or functional impairment could provide useful information for treatment planning.

Several studies have used the Autism Quotient (AQ; Baron‐Cohen, Wheelwright, Skinner, Martin, & Clubley, 2001) or the abbreviated version (AQ‐10; Allison, Auyeung, & Baron‐Cohen, 2012) in individuals with AN, generally showing that those with AN score significantly higher than HCs (Westwood et al., 2016). The AQ is a 50 item self‐report questionnaire assessing five domains: social skills; attention switching; attention to detail; communication; and imagination. In the original validation study, the AQ demonstrated reasonable face validity, with 80% of individuals with ASD scoring above the cut‐off of 32, compared to 2% of HCs (Baron‐Cohen et al., 2001). The AQ‐10 has similar sensitivity and specificity to the full version, where a cut‐off of 6 is recommended for screening purposes (Booth et al., 2013). However, there is some evidence to suggest that the AQ performs poorly in predicting an ASD diagnosis in adults with suspected ASD (Ketelaars et al., 2008). For example, in a large sample of adults referred to a national diagnostic service, Ashwood et al. (2016) reported that two‐thirds of those scoring below the AQ cut‐off were “false negatives” who did in fact have ASD (assessed using the ADOS and ADI‐R). Neither version of the AQ correlated with ADOS scores, although weak correlations were found with the ADI‐R. Further, “false positives” (those that scored above the AQ cut‐off but did not receive a formal ASD diagnosis) were more likely to have comorbid general anxiety disorder, suggesting anxiety may inflate AQ scores. Only a few studies have examined associations between self‐report ASD measures such as the AQ and scores on diagnostic interviews in individuals with AN. Generally, there is poor agreement between measures. Rhind et al. (2014) found that AQ‐10 scores did not differ between adolescents with AN who were assigned an ASD diagnosis (using the Development and Well‐being Assessment; Goodman, Ford, Richards, Gatward, & Meltzer, 2000) and those that did not. Sedgewick, Kerr‐Gaffney, Leppanen, and Tchanturia (2019) found that AQ‐10 scores were positively associated with ADOS‐2 (Lord et al., 2012) scores in individuals recovered from AN but not in those with acute AN.

One measure that has been used extensively in individuals with ASD is the social responsiveness scale (SRS; Constantino & Gruber, 2005). The SRS is a 65 item parent‐ or teacher‐rated questionnaire and is often administered as part of a comprehensive diagnostic assessment of ASD in those between the ages of 4 and 18 (Duku et al., 2013). More recently, an adult self‐report version was developed (SRS‐2; Constantino & Gruber, 2012). Like the original SRS, the SRS‐2 comprises five subscales based on diagnostic criteria for ASD: social motivation; social awareness; social cognition; social communication; and restricted interests and repetitive behaviour. Total scores can be converted into *T*‐scores in order to give an indication of severity of an individual's symptoms. *T*‐scores falling within the mild, moderate, or severe range suggest clinically significant symptoms with varying degrees of impact on everyday social interactions. Dimensions of the SRS‐2 have been found to correspond to the DSM‐5 criteria domains for ASD (Frazier et al., 2014), and total scores discriminate those with ASD from non‐ASD clinical populations (Takei et al., 2014). The SRS‐2 also shows good concurrent and convergent and concurrent validity, correlating with measures of adaptive behaviour and the ADI‐R in adults with ASD (Chan, Smith, Hong, Greenberg, & Mailick, 2017). Further, experimental evidence suggests that higher scores on the SRS‐2 are associated with reduced social attention in those with ASD, one of the core characteristics of the disorder (Dijkhuis, Gurbuz, Ziermans, Staal, & Swaab, 2019; Hanley et al., 2015; Ketelaars et al., 2017) To date, no study has used the SRS‐2 in individuals with AN.

The primary aim of the current study was to examine associations between scores on the SRS‐2 and scores on an observational diagnostic measure, the ADOS‐2, in adults with AN. Because the SRS‐2 has not yet been used in this clinical population, we also aimed to explore group differences in SRS‐2 scores between individuals currently ill with AN compared to recovered AN and HCs. Finally, the study aimed to examine associations between SRS‐2 scores, ED severity, and functional impairment.

## 2 METHODS

### 2.1 Participants and design

The study was cross‐sectional with three groups: acute AN, recovered AN (REC), and HCs. Ethical approval was obtained from the National Health Service (NHS) Research Ethics Committee (Camberwell St Giles, 17/LO/1960). All participants were required to be between 18 and 55 years old and fluent in English. A history of brain trauma or learning disability was exclusion criteria. HC participants were recruited through a King's College London email circular and posters around campuses. HCs were screened using the Structured Clinical Interview for DSM‐5 Disorders, research version (SCID‐5‐RV; First, Williams, Karg, & Spitzer, 2015), to ensure they did not meet criteria for any psychiatric disorders. They were also required to have a body mass index (BMI) between 19 and 27.

In addition to the university advertisements, participants with AN or REC were recruited through online advertisements (B‐eat, call for participants, MQ mental health). Participants with AN were also recruited through two specialist NHS ED services in London. AN and REC were screened using the SCID‐5‐RV to confirm a current or past diagnosis of AN. Participants with AN were required to have a BMI ≤18.5 and REC participants a BMI between 19 and 27. Further, REC participants were required to have maintained a BMI within this range for at least 1 year prior to testing.

### 2.2 Procedure and materials

Participants attended a testing session as part of a wider study on socio‐emotional processing at the Institute of Psychiatry, Psychology & Neuroscience; however, where participants were inpatients (*N* = 11), testing took place at their place of treatment. Written informed consent was obtained, and the following measures were administered in order:

The Wechsler Abbreviated Scale of Intelligence – second edition (WASI‐II; Wechsler, 2011) was used to estimate IQ. The two subtest version was used (vocabulary and matrix reasoning).

The Autism Diagnostic Observation Schedule – second edition (ADOS‐2; Lord et al., 2012) is a standardised semi‐structured observational interview for the assessment of ASD. Module 4 is intended for use with verbally fluent adults and thus was used in this study. The interview includes a range of questions and activities designed to evoke behaviours and cognitions associated with ASD. Items are scored on a scale of 0–3, with higher scores indicating more autistic behaviour. The revised algorithm, which was designed to more closely reflect the DSM‐5 criteria for ASD was used for scoring (Hus & Lord, 2014). The algorithm has two subscales: social affect and restrictive and repetitive behaviours, and total scores of 8 or more indicate possible ASD. The interview was administered by the first author, who met requirements for ADOS‐2 research reliability.

The SRS‐second edition, adult self‐report form (SRS‐2; Constantino & Gruber, 2012) is a 65‐item questionnaire assessing symptoms associated with ASD, with higher scores indicating more autistic symptoms. There are five sub‐scales: social awareness (ability to recognise social cues, for example, item 7, “I am usually aware of how others are feeling”), social cognition (interpreting social behaviour, for example, item 48, “I have a good sense of humor and can understand jokes”), social communication (reciprocal communication in social situations, for example, item 16, “I avoid eye contact or am told that I have unusual eye contact”), social motivation (motivation to participate in social interactions, for example, item 6, “I would rather be alone than with others”), and restrictive interests and repetitive behaviour (circumscribed interests and stereotypy, for example, item 24, “I have more difficulty than others with changes in my routine”). Respondents indicate their agreement with each item on a four‐point Likert scale, rating their behaviour over the past 6 months. The sum of all items is calculated to provide a total score (max 195). *T*‐scores are interpreted as: ≤ 59*T*, within normal limits; 60–65 *T*, mild; 66–75 *T*, moderate; ≥ 76*T* severe range. Cronbach's alpha was 0.97.

The Eating Disorder Examination Questionnaire (EDE‐Q; Fairburn & Beglin, 1994) was used to measure severity of ED psychopathology. Global scores are calculated by averaging responses across items, with higher scores indicating more severe symptoms (max 6). HCs with a score of >2.7 were excluded to ensure those with possible sub‐threshold ED symptoms were not included (Lang et al., 2016). Cronbach's alpha was 0.98.

The Work and Social Adjustment Scale (WSAS; Mundt, Marks, Shear, & Greist, 2002) is a brief measure of functional impairment in five domains: work, home management, social leisure, private leisure, and ability to form and maintain close relationships. Scores range from 0 to 40, with a score of 20 or more indicating clinical significance. Cronbach's alpha was 0.93.

Participants' heights and weights were taken to calculate BMI (weight/height^2^).

### 2.3 Analytic plan

Histograms and Q‐Q plots were inspected to check for normal distributions. Where data were positively skewed, a logarithmic transformation was applied. Homogeneity of variances was assessed using Levene's test. Group differences on continuous variables were examined using one‐way ANOVAs and Tukey's post‐hoc tests, or Welch's ANOVA with Games‐Howell post‐hoc tests where the assumption of homogeneity was violated. Group differences on dichotomous variables were assessed using chi‐squared tests of homogeneity (or Fisher's exact test where the sample size assumption was not met). Zero‐order correlations were calculated to examine associations between SRS‐2 and ADOS‐2 scores, ED severity (BMI, EDE‐Q scores, illness length), and functional impairment (WSAS scores). Where significant correlations were found, regression analyses were run to examine whether SRS‐2 scores predicted ADOS‐2 scores, ED severity, and functional impairment.

## 3 RESULTS

### 3.1 Demographic information

One hundred and fifty‐three participants were recruited. Out of 51 HCs, 5 were excluded based on their EDE‐Q scores, and 1 REC participant was excluded as their BMI was above 27. A further 2 HCs, 1 REC, and 2 AN did not complete the SRS‐2 and were thus excluded from analyses. Thus, data from 44 HCs, 49 REC, and 49 AN are presented here. Demographic information is presented in Table 1. Groups were of similar age, sex, and IQ. Over half of individuals with AN (53.5%) reported having at least one comorbid psychiatric disorder, compared to 38.8% of those in the REC group. The most common were depressive disorders (32.6% of AN, 18.4% of REC) and anxiety disorders (30.2% of AN, 22.4% of REC).

**TABLE 1:** Mean (SD) demographic information

	AN (*N* = 49)	REC (*N* = 49)	HC (*N* = 44)	Test statistics	*p*‐value	*ηp* ^2^/*d*
Age (years)^†^	27.14 (8.78)	26.0 (8.1)	23.54 (4.59)	F(2, 89.41) = 2.32	.10	.03
% female	93.2	98.0	93.9	Fisher's exact test = 1.46	.63	
BMI	15.72 (1.44)^a^	21.14 (1.91)^b^	21.70 (1.91)^b^	F(2, 140) = 167.81	**<.001**	.71
Years of education	16.14 (3.18)	16.52 (2.62)	16.62 (2.49)	F(2, 132) = 0.36	.70	.01
IQ	110.21 (12.91)	110.16 (10.81)	114.21 (6.82)	F(2, 138) = 2.15	.12	.03
Illness length (years)	7.77 (7.86)	5.40 (5.65)	**—**	t(87.21) = 1.69	.09	.35
% on psychiatric medication	55.1^a^	32.7^b^	**—**	*X* ^2^ = 5.39	**.02**	

*Note:* Different superscripts indicate significant differences between groups, significant *p*‐values are highlighted in bold.

^†^Variable was log transformed for analyses, original values are displayed.

Abbrevitaions: AN, anorexia nervosa; BMI, body mass index; HC, healthy control; IQ, intelligence quotient; REC, recovered anorexia nervosa; SD, standard deviation.

### 3.2 ASD symptoms

Group differences on the SRS‐2 and ADOS‐2 are displayed in Table 2. Generally, SRS‐2 total and subscale scores were significantly higher in both AN and REC compared to HCs. The exception was social awareness, where scores in REC did not significantly differ from that of AN or HC, lying in the middle. AN scored significantly higher than HC on ADOS‐2 total and subscale scores. In addition, a significantly higher proportion of individuals with AN (26.5%) and REC (24.5%) scored above the ADOS‐2 clinical cut‐off compared to HCs (4.5%) (*X* ^2^ = 8.73, *p* = .01).

**TABLE 2:** Mean (SD) total and subscale scores on the SRS‐2 and ADOS‐2

	AN (N = 49)	REC (N = 49)	HC (N = 44)	Test statistics	*p*‐value	*ηp* ^2^
SRS‐2 total	85.29 (32.78)^a^	70.04 (31.97)^a^	39.23 (20.18)^b^	F(2,90.14) = 39.08	**<.001**	.30
Social awareness	8.73 (2.81)^a^	7.59 (3.27)	6.48 (2.45)^b^	F(2,139) = 7.14	**<.001**	.09
Social cognition	13.92 (6.86)^a^	11.08 (6.32)^a^	5.93 (4.83)^b^	F(2,92.03) = 23.51	**<.001**	.23
Social communication	27.24 (11.47)^a^	22.08 (11.88)^a^	12.75 (8.42)^b^	F(2,91.94) = 26.21	**<.001**	.24
Social motivation	19.08 (7.01)^a^	15.98 (6.96)^a^	8.52 (4.03)^b^	F(2,88.78) = 48.78	**<.001**	.33
Restricted interests and repetitive behavior	16.31 (8.40)^a^	13.31 (7.83)^a^	5.55 (3.99)^b^	F(2,85.47) = 41.95	**<.001**	.29
ADOS‐2 total	5.37 (4.49)^a^	4.16 (4.50)	2.70 (2.56)^b^	F(2,88.52) = 6.81	**.002**	.07
ADOS‐2 social affect	4.67 (4.11)^a^	3.71 (3.96)	2.52 (2.40)^b^	F(2, 89.32) = 5.26	.**007**	.06
ADOS‐2 restricted and repetitive behaviors	0.69 (1.02)^a^	0.45 (0.89)	0.18 (0.58)^b^	F(2, 89.71) = 4.86	.**010**	.06

*Note:* Different superscripts indicate significant differences between groups, significant *p*‐values are highlighted in bold.

Abbreviations: ADOS‐2, autism diagnostic observation schedule, second edition; AN, anorexia nervosa; HC, healthy control; REC, recovered anorexia nervosa; SD, standard deviation; SRS‐2, social responsiveness scale, second edition.

**FIGURE 1:**
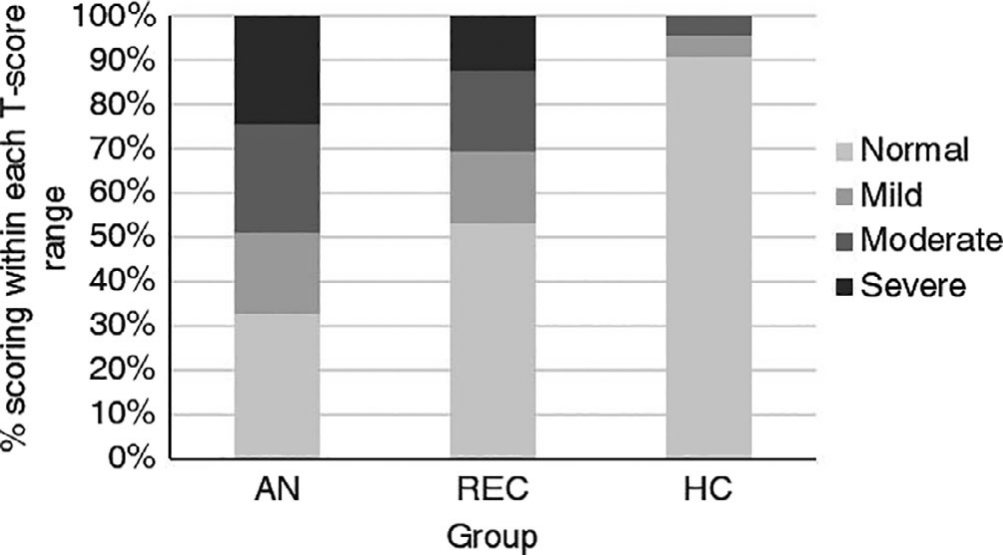
Proportion of participants scoring within each T‐score range on the social responsiveness scale, adult self‐report version (SRS‐2)

The distribution of SRS‐2 T‐scores is displayed in Figure 1. Ninety‐one percent of HCs scored within the “normal” range, compared to 32.7% of participants with AN, and 53.1% of REC. Of the HCs, 4.5% scored within the “mild” range, compared to 18.4% of participants with AN and 16.3% of REC. Similarly, 4.5% of HCs scored within the “moderate” range, compared to 24.5% of participants with AN and 18.4% of REC. Finally, 24.5% of participants with AN and 12.2% of REC scored within the “severe” range, while no HCs did.

### 3.3 Associations between ASD measures

Associations between the SRS‐2 and ADOS‐2 in AN and REC groups are presented in Table 3. SRS‐2 total scores were significantly positively associated with ADOS‐2 total scores and the SA subscale in both groups, but not the RRB subscale.

**TABLE 3:** Correlations between ASD measures

Group	Variables	1	2	3	4
AN	1. SRS‐2 total	**—**			
	2. ADOS‐2 total	.41**	**—**		
	3. ADOS‐2 SA	.39**	.97***	**—**	
	4. ADOS‐2 RRB	.22	.48***	.27	**—**
REC	1. SRS‐2 total	**—**			
	2. ADOS‐2 total	.47***	**—**		
	3. ADOS‐2 SA	.51***	.99***	**—**	
	4. ADOS‐2 RRB	.10	.66***	.53***	**—**

*Note:* ** = *p* < .01; *** = *p* < .001.

Abbreviations: ADOS‐2, autism diagnostic observation schedule, second edition; AN, anorexia nervosa; ASD, autism spectrum disorder; REC, recovered anorexia nervosa; RRB, restricted and repetitive behaviours; SA, social affect; SRS‐2, social responsiveness scale, second edition.

To ascertain whether SRS‐2 scores predicted ADOS‐2 classification (based on the clinical cut‐off of 8) in the whole sample, a binomial logistic regression was run. The model was statistically significant, *X* ^2^(1) = 20.97, *p* < .001. The model explained 22% (Nagelkerke R^2^) of the variance in ADOS‐2 scores and correctly classified 84.5% of cases. The positive predictive value (PPV) was 77.78%, and the negative predictive value (NPV) was 84.96%.

### 3.4 Associations between SRS‐2 scores, ED severity, and functional impairment

In both AN and REC, SRS‐2 total scores were significantly positively correlated with EDE‐Q scores and degree of functional impairment (WSAS score), but not BMI or illness duration (see Table 4).

**TABLE 4:** Correlations between SRS‐2 scores, ED severity, and WSAS scores

Group	Variables	1	2	3	4	5
AN	1. SRS‐2 total	**—**				
	2. EDE‐Q total	.31*	**‐**			
	3. BMI	−.12	.06	**—**		
	4. Illness duration	−.01	−.03	−.14	**—**	
	5. WSAS	.69***	.42**	−.16	.20	**—**
REC	1. SRS‐2 total	**—**				
	2. EDE‐Q total	.46***	**—**			
	3. BMI	−.09	.11	**—**		
	4. Illness duration	−.10	−.01	−.21	**—**	
	5. WSAS	.57***	.59***	−.05	−.09	**—**

AN, anorexia nervosa; BMI, body mass index; ED, eating disorder; EDE‐Q, eating disorder examination questionnaire; REC, recovered anorexia nervosa; SRS‐2, social responsiveness scale, second edition; WSAS, work and social adjustment scale.

* = *p* < .05; ** = *p* < .01; *** = *p* < .001.

Multiple regression analyses were run to examine whether SRS‐2 scores predicted EDE‐Q and WSAS scores (Table 5). Group (acute or recovered AN) and BMI were also entered as covariates. Both models were significant (both *p* < .001). Along with group membership, SRS‐2 total scores significantly added to the prediction of EDE‐Q and WSAS scores.

**TABLE 5:** Multiple regression analysis predicting EDE‐Q and WSAS scores from SRS‐2 scores

	EDE‐Q			WSAS		
	*b*	SE_*b*_	*β*	b	SE_*b*_	*β*
SRS‐2 total covariates	.02	.00	.32***	.17	.02	.53***
BMI	.10	.08	.18	−.16	.42	−.05
Group	−2.44	.50	−.70***	−8.42	2.65	−.40**
Adjusted *R* ^2^	.47			.58		

Abbreviations: BMI, body mass index; EDE‐Q, eating disorder examination questionnaire; SE_*b*_, standard error of the coefficient; SRS‐2, social responsiveness scale, second edition; WSAS, work and social adjustment scale.

*Note:* ** = *p* < .01; *** = *p* < .001; *b*, unstandardized regression coefficient; *β*, standardized coefficient.

## 4 DISCUSSION

The main purpose of the current study was to examine whether scores on a widely used self‐report measure of ASD symptoms, the SRS‐2, were related to scores on a “gold‐standard” diagnostic tool, the ADOS‐2, in individuals with current or past AN. Indeed, there were significant positive correlations between measures, and SRS‐2 scores significantly predicted ADOS‐2 classification. Analyses of group differences revealed that individuals with AN and REC demonstrated significantly higher SRS‐2 total and social cognition, social communication, social motivation, and restrictive interests and repetitive behaviour subscale scores. On the social awareness subscale, individuals with AN scored significantly higher than HCs, while REC showed an intermediate profile and did not significantly differ from either of the other two groups. Finally, ASD traits significantly predicted both severity of ED psychopathology and degree of functional impairment, but not BMI or illness duration.

The significant positive correlations between the SRS‐2 and the ADOS‐2 suggest the SRS‐2 may be a useful tool for assessing ASD traits in individuals with AN both in research and clinical settings. Given the presence of elevated ASD traits in AN is associated with poorer outcomes (Anckarsäter et al., 2012; Nielsen et al., 2015; Wentz, Gillberg, Anckarsäter, Gillberg, & Råstam, 2009), and less improvement during treatment (Stewart, McEwen, Konstantellou, Eisler, & Simic, 2017; Tchanturia, Larsson, & Adamson, 2016), accurate measurement and identification of possible cases is important. Correlations between measures were of medium strength in both AN and REC, and our findings were strengthened by a regression analysis, which showed that SRS‐2 scores significantly predicted ADOS‐2 classification (based on the clinical cut‐off of 8). Studies using the SRS‐2 and the ADOS‐2 in individuals with ASD have demonstrated similar associations (Takei et al., 2014). It must be noted that scoring above the clinical cut‐off on the ADOS‐2 does not provide enough information to receive a diagnosis of ASD. In addition to the assessment of current ASD symptoms, a battery of measures are recommended, in order to obtain information about early developmental history, behavioural problems, functioning at home and in education or employment, comorbidities, and sensory sensitivities (NICE, 2012). Future research would benefit from using the SRS‐2 in individuals with AN who have undergone a full ASD assessment.

Total SRS‐2 scores were significantly higher in individuals with AN and REC compared to HCs, with a large effect size. Indeed, mean scores were similar to those that have been reported in adults with ASD (e.g., Dijkhuis et al., 2019; Maddox & White, 2015; Takei et al., 2014; Walsh, Baxter, Smith, & Braden, 2019). Further, around half of participants with AN scored within the “moderate” or “severe” impairment range, compared to just under one‐third of REC, and only 4.5% of HCs. These findings are in agreement with previous studies demonstrating clinically significant levels of ASD traits in individuals with AN (Westwood & Tchanturia, 2017). Far less work has examined ASD traits in those who have recovered from AN, but generally studies show that elevated ASD traits persist after recovery (Bentz et al., 2017; Sedgewick et al., 2019). These results suggest that high ASD traits seen in those with AN are not a result of starvation or other state effects, although longitudinal research is required. Participants with AN and REC also demonstrated significant impairments across the SRS‐2 subscales, with large effect sizes. The exception was social awareness, where scores in the REC group did not differ from the other two groups, and the magnitude of the effect size for the group difference between AN and HC was also smaller. Females with ASD are reported to show greater awareness of the need for social interaction than males with the disorder (Lai, Lombardo, Auyeung, Chakrabarti, & Baron‐Cohen, 2015); therefore, a possible explanation for our findings may relate to the predominantly female sample included in the study.

In accordance with our findings in recovered AN, the lack of an association between BMI and SRS‐2 scores suggests that elevated ASD symptoms do not merely reflect increased rigidity and social withdrawal that can accompany starvation. Our findings do however suggest an association between increased severity of ED psychopathology and ASD traits in both acute and recovered AN. This finding is in agreement with a previous study demonstrating a positive association between AQ‐10 scores and scores on the EDE‐Q in a large sample of inpatients with AN (Tchanturia, Adamson, Leppanen, & Westwood, 2019). Our study went further by showing that SRS‐2 scores explained a significant proportion of the variance in EDE‐Q scores, however the exact nature of this relationship is not known. Why might ASD symptoms exacerbate ED symptoms? The cognitive interpersonal maintenance model of AN proposes that cognitive rigidity, increased attention to detail, and sensitivity to order are predisposing traits for the illness (Treasure & Schmidt, 2013). Once dieting behaviour is triggered, it is undertaken in a highly meticulous and rigid manner, and the ensuing lack of nutrition further serves to reduce central coherence and increase the narrow focus on food and weight. Thus, it may be that these neuropsychological traits characteristic of ASD perpetuate ED cognitions and behaviours. Another possibility is that the social difficulties associated with ASD lead to isolation, allowing ED cognitions and behaviours to dominate. Indeed, social difficulties have been shown to be an important prognostic factor in AN, predicting poorer outcomes (Franko et al., 2013; Wentz et al., 2009; Zipfel, Löwe, Reas, Deter, & Herzog, 2000). Qualitative work has also emphasised the importance of social support and decreasing isolation as key to recovery (Cockell, Zaitsoff, & Geller, 2004; Federici & Kaplan, 2008; Linville, Brown, Sturm, & McDougal, 2012). Finally, it may be the case that some other factor not measured in this study influences both EDE‐Q and SRS‐2 scores; therefore, replication of our findings in other samples are required.

Similar to past findings in ASD (Chan et al., 2017; Mason et al., 2018), SRS‐2 scores significantly predicted functional impairment, providing further evidence supporting the utility of the instrument in individuals with AN. As well as using the SRS‐2 to identify individuals who may require further ASD assessment, the subscale scores could give valuable information on the specific areas of difficulty with which an individual presents. If appropriate, these could be incorporated into an individualised treatment approach. For example, social skills training might be useful for someone who shows difficulties in the social cognition domain. Group social skills interventions are effective in improving communication, social anxiety, and social functioning in adults with ASD (Spain & Blainey, 2015; Spain, Blainey, & Vaillancourt, 2017). Such interventions could be useful for those with AN, especially those with high ASD traits. On the other hand, cognitive remediation therapy (CRT) may benefit someone with high scores on the restricted interests and repetitive behaviour subscale of the SRS‐2. Preliminary evidence suggests that CRT increases set‐shifting performance in those with AN and ASD traits (Dandil, Smith, Adamson, & Tchanturia, 2019). Whether the SRS‐2 is sensitive to treatment‐related changes in individuals with AN is an interesting question for future research. When using the SRS‐2, clinicians should advise patients that although it is not a diagnostic tool, the SRS‐2 does correlate strongly with other diagnostic measures and could provide indications about some of the challenges they are facing that may not have previously been well thought about in treatment. This might enhance complex formulations and help to guide more efficient and effective treatments. It may also help the patient to think about what sort of additional support they might need as they leave hospital and continue their recovery from AN in the community. The results might also be shared with the person's family and carer support network, with the person's permission and involvement, so that loved ones might make a start on better understanding the person's strengths and challenges.

The study has several limitations. First, only a small proportion of males were included, and as a result, we were unable to examine whether SRS‐2 scores differed by sex, or indeed whether SRS‐2 and ADOS‐2 scores were similarly correlated in males and females. To date, studies exploring ASD traits in individuals with AN have almost all included exclusively female samples, and it is not yet known whether the proportion of males with AN and high ASD traits is similar to that of females. There is evidence to suggest that females with ASD show lower scores compared to males with ASD on diagnostic interviews such as the ADOS‐2, despite showing similar or higher scores on self‐report measures of symptoms (Frazier, Georgiades, Bishop, & Hardan, 2014; Lai et al., 2011; Mason et al., 2018). The scoring algorithms used in diagnostic interviews may be biased towards identification of male‐typical presentations, given the longstanding male predominance in ASD case identification (Lai et al., 2015). Relatedly, females with ASD may use more compensatory strategies, showing better social communication than males, despite similar levels of underlying difficulties and distress (Lai et al., 2017). One might expect then that an even greater proportion of males with AN may score above the ADOS‐2 cut‐off than females with AN; however, this remains a question for future research.

Second, while the ADOS‐2 is recommended for use in diagnostic assessments of ASD, alone it does not provide enough information to give a diagnosis. The current study would benefit from including a group of individuals with AN who hold a confirmed diagnosis of ASD, in order to better test the predictive power of the SRS‐2 as a screening tool. Third, the cross‐sectional design should be noted when interpreting group differences between the acute and REC groups. It is possible that differences in ED or ASD psychopathology contributed to the recovery of the REC group. Research examining ASD traits longitudinally in individuals with AN would provide stronger evidence to delineate state versus trait effects. Finally, a history of psychiatric disorders was an exclusion criteria for HCs; however, comorbidities were allowed in individuals in the AN and REC groups, introducing a potential confound to the results. Relatedly, we were unable to corroborate comorbid psychiatric diagnoses in AN and REC participants via psychiatric interviews, therefore, preventing an analysis of the potential confounding effect of anxiety and depression on ASD symptoms. Although there is evidence to suggest that ADOS‐2 scores are largely unrelated to anxiety and depression in individuals with AN (Sedgewick et al., 2019), it is not yet known whether SRS‐2 scores are influenced by affective symptoms in this population. Indeed, many of the items on the SRS‐2 are also symptoms of disorders such as social anxiety (e.g., lack of eye contact, discomfort in social situations). Investigations into the trajectory of symptoms over time may be useful in clarifying this issue.

## 5 CONCLUSION

Recent evidence has accumulated to suggest an association between AN and ASD, raising important questions for both research and clinical practice. Currently, there is a lack of agreement on which tools should be used to assess ASD in individuals with AN. To our knowledge, this is the first study to use the SRS‐2 in a sample of adults in the acute and recovered stages of AN. In agreement with previous studies showing high ASD traits in those with AN, participants in the acute and recovered stage of AN scored significantly higher on the SRS‐2 compared to age‐ and sex‐matched HCs. Scores on the SRS‐2 significantly predicted ADOS‐2 classification in the whole sample, suggesting the SRS‐2 may be useful in identifying individuals with suspected ASD whose symptoms may benefit from further investigation. Positive associations between SRS‐2 scores, functional impairment, and ED psychopathology further support the utility of the measure within this population. Replications in larger samples are required to confirm the reliability of our results, and future research should employ longitudinal designs in order to examine illness versus trait factors that may influence ASD symptoms in AN.

## CONFLICT OF INTEREST

The authors have no conflicts of interest.

## Data Availability

The data that support the findings of this study are available from the corresponding author upon reasonable request.
